# Retraction: The Use of CT Coronary Angiography and CT Fractional Flow Reserve in the Investigation of Patients With Suspected Coronary Artery Disease

**DOI:** 10.7759/cureus.r19

**Published:** 2020-06-17

**Authors:** Mohammad Fawad Khattak, Sebastian Horne

**Affiliations:** 1 Cardiology, Russells Hall Hospital, Dudley, GBR

The authors of this article discovered that key errors had been made in matching heart-flow analysis data with the correct patients. As a result, the data contained within this study is incorrect and alters the findings and results. Based on this information, the authors have requested retraction and, after careful examination, Cureus editors have agreed.

